# The comparative risk of developing postoperative complications in patients with distal radius fractures following different treatment modalities

**DOI:** 10.1038/srep15318

**Published:** 2015-11-09

**Authors:** Wen-Jun Qiu, Yi-Fan Li, Yun-Han Ji, Wei Xu, Xiao-Dong Zhu, Xian-Zhong Tang, Huan-Li Zhao, Gui-Bin Wang, Yue-Qing Jia, Shi-Cai Zhu, Feng-Fang Zhang, Hong-Mei Liu

**Affiliations:** 1 Department of Orthopedic Surgery, Tongren Hospital, Shanghai Jiaotong University School of Medicine, Shanghai 200336, P.R. China

## Abstract

In this study, we performed a network meta-analysis to compare the outcomes of seven most common surgical procedures to fix DRF, including bridging external fixation, non-bridging external fixation, K-wire fixation, plaster fixation, dorsal plating, volar plating, and dorsal and volar plating. Published studies were retrieved through PubMed, Embase and Cochrane Library databases. The database search terms used were the following keywords and MeSH terms: DRF, bridging external fixation, non-bridging external fixation, K-wire fixation, plaster fixation, dorsal plating, volar plating, and dorsal and volar plating. The network meta-analysis was performed to rank the probabilities of postoperative complication risks for the seven surgical modalities in DRF patients. This network meta-analysis included data obtained from a total of 19 RCTs. Our results revealed that compared to DRF patients treated with bridging external fixation, marked differences in pin-track infection (PTI) rate were found in patients treated with plaster fixation, volar plating, and dorsal and volar plating. Cluster analysis showed that plaster fixation is associated with the lowest probability of postoperative complication in DRF patients. Plaster fixation is associated with the lowest risk for postoperative complications in DRF patients, when compared to six other common DRF surgical methods examined.

Distal radius fracture (DRF) accounts for 1/6^th^ of all fracture-related emergency room visits and is a common bone fracture of the upper limb occurring in the distal end of the radius^1^. DRFs have bimodal distribution, with the first peak observed in pediatric patients, where DRF constitutes approximately 25% of all fractures, and the second DRF peak is seen in the elderly population, constituting 18% of all fractures in this age group^2^. DRF incidence is estimated at 26 per 10,000 person-years and it accounts for 72% of all fractures of the forearm and 8 ~ 17% of all fractures of the extremities^3^. Multiple risk factors for DRFs have been identified, such as gender, environmental conditions (ice and snow), sports activities, vitamin D deficiency, osteoporosis and certain medication (glucocorticosteroids)^4^. The most common complications of DRFs include tendon rupture, arthrosis, chronic regional pain syndrome (CRPS), neurologic compromise, malunion, nonunion, ulnar impaction, stiffness, loss of rotation and, in rare occasion, compartment syndrome^5^. Other notable complications of DRFs are pin-track infection (PTI) and carpal tunnel syndrome (CTS)^6^. Thus, careful attention must be paid to the initial presentation and pattern of injury to ensure successful surgical outcomes and to avoid postoperative complications. Surgical interventions for DRFs include bridge plating, percutaneous Kirschner wire (K-wire) fixation, closed reduction and cast immobilization, fixation with volar or dorsal plates^7^. Study showed that specific treatment approaches are generally chosen based on DRF injury features and the individual surgeon’s experience with various approaches^8^.

Bridging external fixation is a popular method used with static fixators to bridge the wrist and immobilize the wrist joint and the fracture, with fracture reduction maintained by ligametotaxis^9^. Non-bridging external fixation of DRFs is a general technique associated with lower risk of dorsal malunion, compared to bridging external fixation^10^. K-wire fixation is a minimally invasive procedure for DRF, between conservative treatment and open reduction internal fixation (ORIF), which involves wires passing through the skin and into the bone to hold the fracture in its correct anatomical position^11^. Plaster fixation with closed reduction is traditionally performed in older DRF patients where exploration is necessary to assess soft tissue injury, if the fracture is transverse or stable on reduction^12^. Dorsal plating enables direct exposure and reconstruction of the joint with a capsular incision, but requires dissection of extensor retinaculum and plate positioning under this tendon, often resulting in tendonitis or tendon rupture^13^. Volar plating is increasingly used in elderly DRF patients since it provides better reduction, fracture stability and early mobilization. In addition, compared to dorsal plating, volar plating has better soft-tissue coverage and less tendon irritation^14^. Dorsal and volar plating with conventional plates report good clinical outcomes in younger patients sustaining variety of complex fractures^15^. Although DRFs are routinely treated with both surgical and non-surgical approaches, the best approach for DRF treatments remains hotly debated^14^,^16^. Thus, studies comparing different treatment approaches for DRF are extremely helpful for both patients and surgeons to understand the risks.

Meta-analysis framework allows pooling outcomes of homogeneous studies on the same topic and but comparisons between more than two interventions are not possible^17^. On the other hand, a network meta-analysis can indirectly compare three or more interventions and can simultaneously integrate both direct and indirect comparisons of multiple interventions^18^,^19^. In this study, we use network meta-analysis to compare the risks of postoperative complications in DRF patients treated with bridging external fixation, non-bridging external fixation, K-wire fixation, plaster fixation, dorsal plating, volar plating, and dorsal and volar plating.

## Methods and Materials

### Search strategy

PubMed, EBSCO and Cochrane Library databases were exhaustively searched (last updated search, May 2015) to identify published randomized clinical trials (RCTs) relevant to seven common surgical interventions in DRF patients. Search terms used for retrieving relevant literature from these databases included combinations of the following keywords and MeSH terms: DRF, bridging external fixation, non-bridging external fixation, K-wire fixation, plaster fixation, dorsal plating, volar plating, and dorsal and volar plating.

### Inclusion and exclusion criteria

Studies were selected for incorporation into this network meta-analysis if they conformed to the following inclusion criteria: (1) study type: RCTs; (2) interventions: bridging external fixation, non-bridging external fixation, K-wire fixation, plaster fixation, dorsal plating, volar plating, and dorsal and volar plating; (3) study subjects: patients clinically or radiologically confirmed as DRF; (4) study outcomes: incidence of CTS, CRPS and PTI rate in DRF patients. Studies were excluded if they (1) lacked data integrity; (2) were not RCTs; (3) were duplicate studies; or (4) involved complex intervention strategies.

### Data extraction

Two investigators independently extracted the required data using a standard data collection form. The following information was collected from the selected studies: first author, publication year, country, ethnicity, language, disease, interventions, age, gender, adverse outcomes and number of research subjects. Any disagreements between the two investigators during study selection or data collection were resolved by discussion, re-examination of the data or consulting other investigators.

### Statistical analysis

R 3.2.0 software, an open source statistical program, was used to generate the graphical output of the network diagram. Each node represents an intervention, while the node size represents sample size and the width of the connecting line between each node represents the number of studies reporting the comparison. The gemtc installation package of the R software provided a comprehensive set of predictive tools to conduct network meta-analysis in a Bayesian setting.There were four common outcomes for input of Arm- or contrast-level network data: binary, continuous, count or survival. As first described by Lu and Ades, it models relative effects (e.g., log-odds ratio) by setting a generalized linear model (GLM) under Bayesian framework by connecting to JAGS, WinBUGS or OpenBUGS^20^, which was subsequently extended by others^21^,^22^. One of the most important feature of this package is its ability to model inconsistency^22^,^23^. The software provides modeling flexibility since users can specify different likelihood and link functions, several Markov-Chain Monte-Carlo (MCMC) sampling options and priors for hyper parameters. Rankograms was utilized for plotting the estimates of rank probabilities. The surface under the cumulative ranking curve (SUCRA), a simple transformation of the mean rank, allows organization of the treatments by hierarchy, both for variance and location of all relative treatment effects^24^. Higher SUCRA value means a better ranking of the treatment. Multivariate analysis was applied for multiple outcomes in order to explain the correlation between outcomes. Cluster analysis, as an exploratory data mining technique, was used for grouping objects based on their features, with low degree of association between members of different groups and high degree of association between members of the same group^25^. Using clusterank command, clustered ranking plots can be obtained in STATA program. Outcome1 and outcome2 became the data variables containing the SUCRA scores for all treatments in this network. The different colors correspond to the estimated clusters and were utilized for grouping the treatments according to their similarity for both outcomes.

## Results

### Baseline characteristics of included studies

Electronic database search and manual searches retrieved a total of 1249 articles. After excluding 227 duplicate studies, 36 letters and reviews, 12 non-human studies and 209 studies irrelevant to DRF, 765 studies remained for full-text evaluation. Of the 765 studies, 289 articles were excluded since they were not RCTs or were irrelevant to PTI, CRPS and CTS, and 11 studies were eliminated for lack of sufficient data or for having incomplete or weakly correlative data. Finally, 19 RCTs met our stringent inclusion/exclusion criteria and these 19 studies were selected for inclusion in our network meta-analysis^9^,^10^,^14^,^26^,^27^,^28^,^29^,^30^,^31^,^32^,^33^,^34^,^35^,^36^,^37^,^38^,^39^,^40^,^41^. The 19 studies contained a combined total of 1,805 patients who underwent various surgical treatments for DRF (496 patients with bridging external fixation; 525 patients with non-bridging external fixation; 243 patients with K-wire fixation; 84 patients with plaster fixation; 71 patients with dorsal plating; 128 patients with volar plating; and 258 patients with dorsal and volar plating). With respect to the outcome indictors, 12 studies reported the incidence of CTS, 19 studies reported PTI rate, and 17 studies reported the incidence of CRPS. Table 1 shows the baseline characteristics for included studies.

### Evidence network

**Figure 1:**
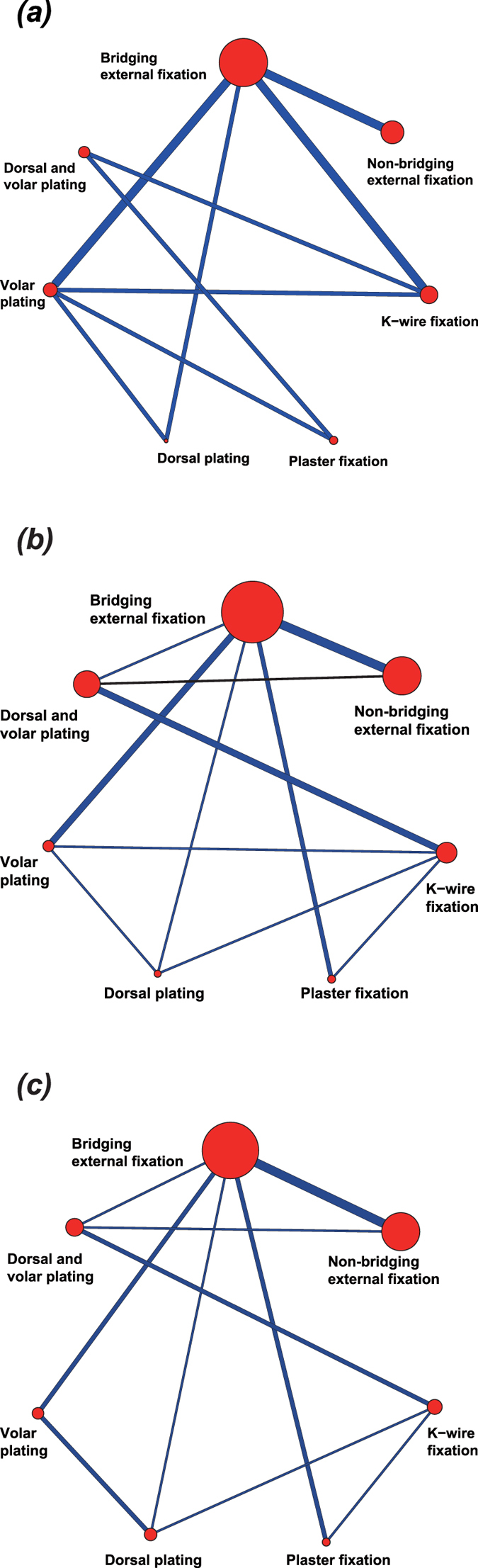
Evidence Networks for the seven surgical treatment modalities. (**a**) carpal tunnel syndrome; (**b**) pin-track infection; (**c**) complex regional pain syndrome).

This study included 7 most common surgical treatments for DRF: bridging external fixation, non-bridging external fixation, K-wire fixation, plaster fixation, dorsal plating, volar plating, and dorsal and volar plating. The highest incidence of CTS was observed in patients treated with bridge external fixation. In this network meta-analysis, majority of the studies showed direct comparisons for bridge external fixation vs. volar plating, bridge external fixation vs. K-wire fixation and bridging external fixation vs. non-bridging external fixation (Fig. 1a). With respect to PTI rate, patients treated with bridge external fixation, non-bridging external fixation and dorsal and volar plating showed the highest rates. In this network meta-analysis, most of the studies showed direct comparisons for bridge external fixation vs. volar plating, dorsal and volar plating vs. K-wire fixation and bridging external fixation vs. non-bridging external fixation (Fig. 1b). The incidence of CPRS was highest in patients treated with bridge external fixation and non-bridging external fixation. In this network meta-analysis, majority of studies showed direct comparison for bridging external fixation vs. non-bridging external fixation (Fig. 1c).

### Test results of inconsistency

**Figure 2:**
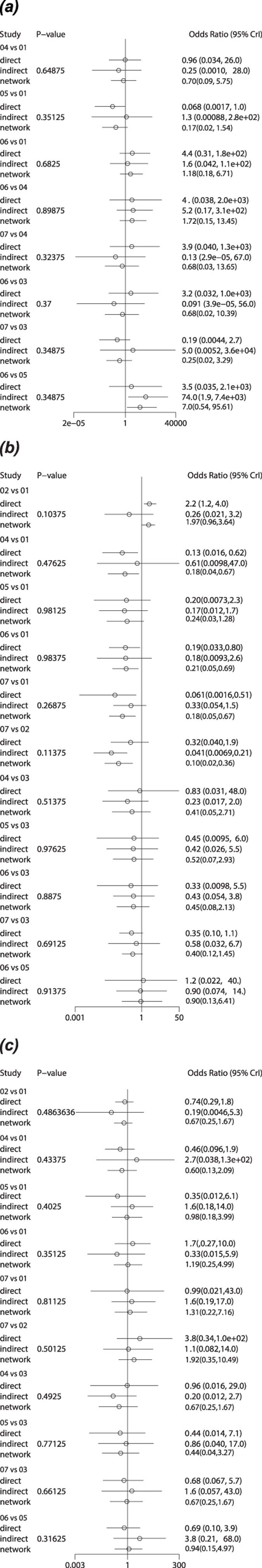
Inconsistency test for the seven treatment modalities. (**a**) carpal tunnel syndrome; (**b**) pin-track infection; (**c**) complex regional pain syndrome; 01: bridging external fixation; 02: non-bridging external fixation; 03: K-wire fixation; 04: plaster fixation; 05: dorsal plating; 06: volar plating; 07: dorsal and volar plating).

Test results of inconsistency for the three adverse outcomes showed that *P* values for all the direct and indirect comparisons were more than 0.05, indicating the results were consistent between direct and indirect comparisons (Fig. 2a–c). Network meta-analysis can be merged, thus the effect sizes of direct and indirect comparisons can be combined using the consistency model.

### The incidence of CTS

**Figure 3:**
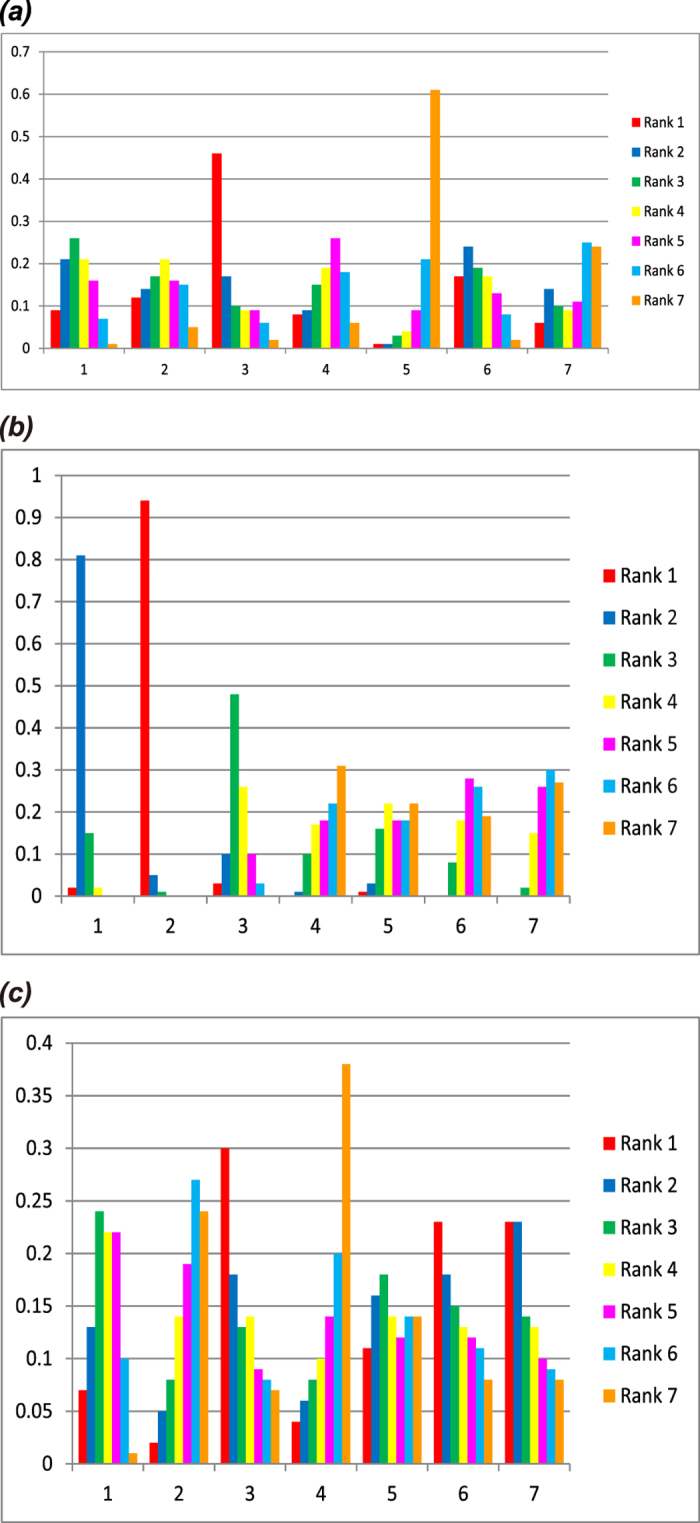
Probability ranking plots for the seven treatment modalities. (**a**) carpal tunnel syndrome; (**b**) pin-track infection; (**c**) complex regional pain syndrome; 1: bridging external fixation; 2: non-bridging external fixation; 3: K-wire fixation; 4: plaster fixation; 5: dorsal plating; 6: volar plating; 7: dorsal and volar plating).

The results of network meta-analysis suggested no statistically significant difference in the incidence of CTS in DRF patients treated with non-bridging external fixation, K-wire fixation, plaster fixation, dorsal plating, volar plating, and dorsal and volar plating when compared to patients treated with bridging external fixation (non-bridging external fixation: OR = 0.86, 95% CI = 0.17 ~ 4.85; K-wire fixation: OR = 1.82, 95% CI = 0.07 ~ 52.85; plaster fixation: OR = 0.70, 95% CI = 0.09 ~ 5.75; dorsal plating: OR = 0.17, 95% CI = 0.02 ~ 1.54; volar plating: OR = 1.18, 95% CI = 0.18 ~ 6.71; dorsal and volar plating: OR = 0.46, 95% CI = 0.02 ~ 13.66) (Table 2a). Dorsal plating ranked seventh with the highest probability among the seven DRF treatments, suggesting the lowest risk of CTS in patients treated with dorsal plating (Fig. 3a). The highest SUCRA value of 89.4% further confirmed that dorsal plating ranked at the top, with the lowest adverse outcome (Table 3).

### PTI rate

The results of network meta-analysis showed statistically significant differences in PTI rate when DRF patients treated with plaster fixation, volar plating, and dorsal and volar plating were compared with patients treated with bridging external fixation (plaster fixation: OR = 0.18, 95% CI = 0.04 ~ 0.67; volar plating: OR = 0.21, 95% CI = 0.05 ~ 0.69; dorsal and volar plating: OR = 0.18, 95% CI = 0.05 ~ 0.67). However, such differences were not found in DRF patients treated with non-bridging external fixation, K-wire fixation and dorsal plating (non-bridging external fixation: OR = 1.97, 95% CI = 0.96 ~ 3.64; K-wire fixation: OR = 0.43, 95% CI = 0.11 ~ 1.85; dorsal plating: OR = 0.24, 95% CI = 0.03 ~ 1.28) (Table 2b). Plaster fixation ranked seventh with the highest probability among the seven DRF treatments, indicating the least risk of PTI in DRF patients treated with plaster fixation (Fig. 3b). The highest SUCRA value of 75.3% further confirmed that the lowest risk for PTI with plaster fixation (Table 3).

### The incidence of CRPS

The results of network meta-analysis revealed no statistically significant differences in the incidence of CRPS in DRF patients treated with non-bridging external fixation, K-wire fixation, plaster fixation, dorsal plating, volar plating, and dorsal and volar plating when compared to patients treated with bridging external fixation (non-bridging external fixation: OR = 0.67, 95% CI = 0.25 ~ 1.67; K-wire fixation: OR = 1.35, 95% CI = 0.23 ~ 10.08; plaster fixation: OR = 0.60, 95% CI = 0.13 ~ 2.09; dorsal plating: OR = 0.98, 95% CI = 0.18~3.99; volar plating: OR = 1.19, 95% CI = 0.25 ~ 4.99; dorsal and volar plating: OR = 1.31, 95% CI = 0.22 ~ 7.16) (Table 2c). Plaster fixation ranked seventh with the highest probability among the seven DRF treatments, indicating the lowest risk for CRPS in DRF patients treated with plaster fixation (Fig. 3c). SUCRA plots further confirmed that plaster fixation had the highest SUCRA value at 77.3% (Table 3).

### Cluster analysis

**Figure 4:**
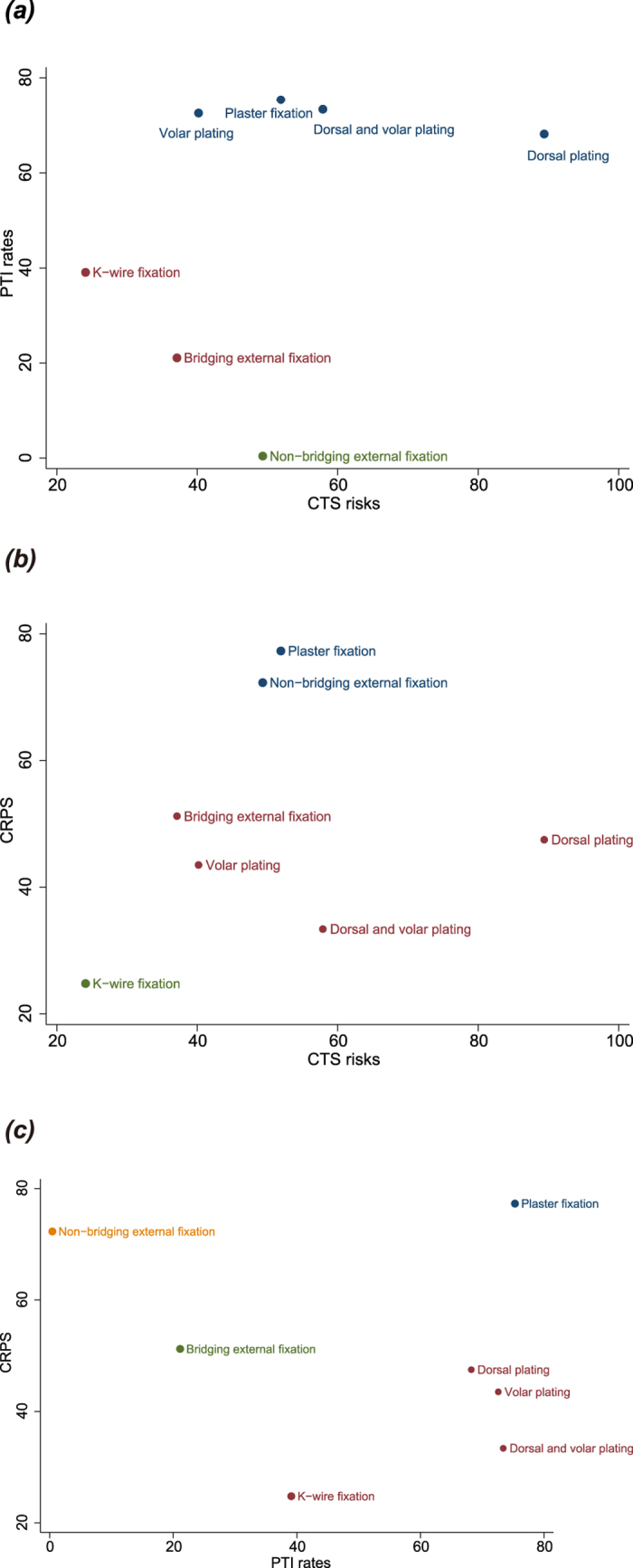
Cluster analysis plots for the seven treatment modalities. (**a**) carpal tunnel syndrome; (**b**) pin-track infection; (**c**) complex regional pain syndrome).

The results of cluster analysis for CTS and PTI rate showed significantly better outcomes for plaster fixation, dorsal plating, volar plating, and dorsal and volar plating in DRF patients (Fig. 4a). The results of cluster analysis for the incidence of CTS and CRPS revealed that plaster fixation and non-bridging external fixation were associated with the best outcomes had better curative effect for DRF patients (Fig. 4b). Cluster analysis for PTI rate and CRPS incidence indicated that plaster fixation had the best outcome in DRF patients (Fig. 4c). Taken together, plaster fixation overall is associated with the lowest risk for postoperative complications in DRF patients.

## Discussion

The best treatment choice for DRFs remains a topic of intense debate. In this study, we compared the risk of postoperative complications in patients who underwent seven different surgical approaches to treat DRF. Our network meta-analysis consisted of 1805 DRF patients pooled from 19 RCTs that reported the risk of CTS, PTI rate and CRPS in DRF patients following various surgical interventions. Our results showed that plaster fixation offered the highest probability for avoiding postoperative complications, compared to six other treatment modalities.

Although, DRF patients treated with dorsal plating carried the lowest risk of CTS, the risk for CRPS and PTI was the lowest in DRF patients treated with plaster fixation. Dorsal plating is a well-established treatment for DRF with several advantages, including ease of exposure, visualization of the articular surface and the biomechanical advantage of placing the plate as a dorsal buttress^42^. However, dorsal plating is associated with extensor tendon complications, which allowed the volar locking plating approach to gain more acceptance^43^. On the other hand, plaster fixation can be tailored according to the fracture features and is suitable for all types of fracture fixation and external fixation, with little adverse reactions^44^. Plaster bandage is a commonly used material in plaster fixation, containing dehydrated calcium sulfate powder that can be easily molded after absorbing water and gradually crystallizes to harden in a short time to maintain the original shape^45^. Also, plaster fixation ensures a relatively uniform pressure on the body’s surface and achieves fracture fixation to a certain extent by limiting muscle contraction^46^.

Interestingly, bridging and non-bridging external fixation, K-wire fixation, dorsal plating, volar plating, and dorsal and volar plating, are described as unsuitable for unstable DRFs and these methods are often associated with poor outcomes. Open reduction and internal fixation was widelyused to fix unstable DRFs in the past, but significant complications have been reported recently, such as rupture of tendons, CTS and CRPS^30^,^47^. Closed reduction and external fixation has also been widely used to treat unstable DRFs for several decades andcomplications include loss of reduction, PTI and stiffness^48^. Notably, the longer the K-wires were left protruding, the greater was the incidence of pin-tract infection, thus it is recommended that K-wires should be buried beneath the skin to reduce infection rate^49^. Similarly, external fixation group (bridging or non-bridging) contributes to early motion of the wrist and pin loosening and is associated with a higher risk of PTI^50^. More recently, angle-stable constructs are available for both dorsal and volar plating approaches, offering sufficient stable fixation to allow early mobilization. However, while these two plating techniques appear to show good results in the short term, a definite high risk of tendonitis, tendon rupture and hardware irritation is reported^51^. Based on the above discussion, plaster fixation carries the lowest risk of postoperative complications, compared to the other six modalities in DRF treatment.

The major merits of this network meta-analysis are: first, due to the absence of head-to-head trials for all the surgical interventions, indirect comparisons were obtained through network analysis. Second, we used consistent measurements across different studies and synthesized the data from selected studies within a single network meta-analysis, avoiding potential selection bias. Third, this updated network meta-analysis provides new insights to address the debate on the best approach by synthesizing the existing evidence and revealing important results related to the clinical care of DRF patients. Our study also has limitations. First, only 19 RCTs were enrolled in our study, the relatively small number of included studies increase the uncertainty of our conclusions. Second, incomplete data existed in some studies, which might bias our results.

In conclusion, our network meta-analysis provides strong evidence that plaster fixation is associated with the lowest risk for postoperative complications in DRF treatment, compared to the six other treatment approaches. However, future RCTs that are better designed and containing larger sample size will be needed to confirm our findings and begin testing clinical applications.

## Additional Information

**How to cite this article**: Qiu, W.-J. *et al.* The comparative risk of developing postoperative complications in patients with distal radius fractures following different treatment modalities. *Sci. Rep.* **5**, 15318; doi: 10.1038/srep15318 (2015).

## Figures and Tables

**Table 1:** The baseline characteristics for included studies.

First author	Year	Country	Intervention		Total	Sample size		Gender (M/F)		Age (years)	
			A1	A2		A1	A2	A1	A2	A1	A2
McQueen MM	1996	UK	Plaster	EF (B)	60	30	30	2/28	4/26	64 ± 14.5	63 ± 11.6
McQueen MM	1998	UK	EF (NB)	EF (B)	60	30	30	3/27	2/28	62 ± 14	61 ± 13
Krishnan J	2003	Australia	EF (NB)	EF (B)	60	30	30	12/18	7/23	58 (18–82)	55 (19–83)
Grewal R	2005	Canada	D	K–wire	62	29	33	12/17	12/21	46 ± 2.7	45 ± 2.7
Kreder HJ	2005	USA	V + D	EF (NB)	179	91	88	59/32	50/38	39 (20–81)	40 (20–78)
Westphal T	2005	Germany	V + D	K–wire	131	54	77	26/28	31/46	59.5 ± 15.8	60.6 ± 15.3
Wright TW	2005	USA	V	EF (B)	32	21	11	11/10	3/8	50.1 (19–74)	50 (21–64)
Atroshi I	2006	Sweden	EF (NB)	EF (B)	38	19	19	3/16	4/15	70 (55–86)	71 (57–84)
Egol K	2008	USA	V	EF (B)	88	44	44	19/25	22/22	52.2 (19–87)	49.9 (18–78)
Hayes AJ	2008	Canada	EF (NB)	EF (B)	588	358	230	47/311	71/159	64	58
Leung F	2008	Taiwan, China	V + D	K-wire	144	70	74	85/52		42 (17–60)	
Abramo A	2009	Sweden	D	EF (B)	50	26	24	14/36		48 (20–65)	
Schmelzer–Schmied N	2009	Germany	V	K-wire	30	15	15	NR		60 (50–70)	
Xu GG	2009	Singapore	V + D	K-wire	30	16	14	9/7	9/5	41.8 (21–56)	45.3 (35–55)
Aktekin CN	2010	Turkey	Plaster	EF (B)	46	24	22	5/19	9/13	71.2 ± 5.2	69.8 ± 4.5
Wong TC	2010	China	K-wire	Plaster	60	30	30	6/24	5/25	70 (66–76)	71 (65–76)
Chappuis J	2011	Belgium	V	D	31	15	16	2/13	2/14	71.73 ± 13.6	71.69 ± 11.2
Grewal R	2011	Canada	V + D	EF (B)	53	27	26	6/20	6/18	58 ± 9.9	53.8 ± 11.7
Wilcke MK	2011	Sweden	V	EF (B)	63	33	30	30/25	33/23	55 (20–69)	56 (21-69)

A, treatment; M, male; F, female; NR, not reported; Plaster, plaster fixation; EF (B), bridging external fixation; EF (NB), non-bridging external fixation; D, dorsal plating; V, volar plating; D + V, dorsal and volar plating; K-wire, K-wire fixation.

**Table 2:** Odds ratio and 95% confidence interval of seven treatment modalities under three end indicators.

(a)						
**Bridging external fixation**	0.86 (0.17, 4.85)	1.82 (0.07, 52.85)	0.70 (0.09, 5.75)	0.17 (0.02, 1.54)	1.18 (0.18, 6.71)	0.46 (0.02, 13.66)
1.16 (0.21, 5.73)	**Non-bridging external fixation**	2.05 (0.06, 98.55)	0.79 (0.06, 10.91)	0.19 (0.01, 3.05)	1.34 (0.11, 14.26)	0.53 (0.01, 22.71)
0.55 (0.02, 13.49)	0.49 (0.01, 17.04)	**K-wire fixation**	0.37 (0.02, 8.71)	0.09 (0.00, 3.87)	0.68 (0.02, 10.39)	0.25 (0.02, 3.29)
1.42 (0.17, 11.23)	1.26 (0.09, 17.88)	2.72 (0.11, 66.07)	**Plaster fixation**	0.24 (0.01, 4.57)	1.72 (0.15, 13.45)	0.68 (0.03, 13.65)
5.97 (0.65, 58.68)	5.24 (0.33, 86.17)	11.33 (0.26, 606.17)	4.10 (0.22, 82.99)	**Dorsal plating**	7.00 (0.54, 95.61)	2.75 (0.05, 142.87)
0.85 (0.15, 5.61)	0.75 (0.07, 9.15)	1.47 (0.10, 40.76)	0.58 (0.07, 6.60)	0.14 (0.01, 1.85)	**Volar plating**	0.42 (0.02, 12.08)
2.20 (0.07, 60.60)	1.88 (0.04, 75.01)	3.96 (0.30, 59.50)	1.47 (0.07, 30.95)	0.36 (0.01, 19.66)	2.40 (0.08, 60.90)	**Dorsal and volar plating**
(b)						
**Bridging external fixation**	1.97 (0.96, 3.64)	0.43 (0.11, 1.85)	0.18 (0.04, 0.67)	0.24 (0.03, 1.28)	0.21 (0.05, 0.69)	0.18 (0.05, 0.67)
0.51 (0.27, 1.04)	**Non-bridging external fixation**	0.23 (0.05, 1.03)	0.09 (0.02, 0.43)	0.12 (0.02, 0.73)	0.11 (0.02, 0.40)	0.10 (0.02, 0.36)
2.31 (0.54, 9.16)	4.42 (0.97, 19.43)	**K-wire fixation**	0.41 (0.05, 2.71)	0.52 (0.07, 2.93)	0.45 (0.08, 2.13)	0.40 (0.12, 1.45)
5.64 (1.48, 28.17)	10.98 (2.35, 60.74)	2.42 (0.37, 18.24)	**Plaster fixation**	1.34 (0.11, 13.86)	1.20 (0.15, 7.95)	0.97 (0.15, 7.64)
4.17 (0.78, 33.19)	8.54 (1.36, 66.04)	1.93 (0.34, 14.36)	0.75 (0.07, 8.95)	**Dorsal plating**	0.90 (0.13, 6.41)	0.77 (0.13, 7.16)
4.88 (1.45, 21.76)	9.29 (2.48, 47.31)	2.23 (0.47, 13.11)	0.83 (0.13, 6.64)	1.12 (0.16, 7.91)	**Volar plating**	0.92 (0.15, 5.44)
5.42 (1.49, 22.20)	10.47 (2.76, 46.52)	2.49 (0.69, 8.18)	1.03 (0.13, 6.88)	1.29 (0.14, 7.91)	1.09 (0.18, 6.84)	**Dorsal and volar plating**
(c)						
**Bridging external fixation**	0.67 (0.25, 1.67)	1.35 (0.23, 10.08)	0.60 (0.13, 2.09)	0.98 (0.18, 3.99)	1.19 (0.25, 4.99)	1.31 (0.22, 7.16)
1.50 (0.60, 4.04)	**Non-bridging external fixation**	2.00 (0.30, 15.25)	0.89 (0.15, 4.63)	1.49 (0.23, 8.09)	1.75 (0.29, 9.67)	1.92 (0.35, 10.49)
0.74 (0.10, 4.43)	0.50 (0.07, 3.36)	**K-wire fixation**	0.44 (0.04, 3.27)	0.71 (0.08, 4.54)	0.86 (0.08, 6.22)	0.94 (0.15, 4.97)
1.66 (0.48, 7.98)	1.12 (0.22, 6.52)	2.29 (0.31, 22.72)	**Plaster fixation**	1.71 (0.22, 12.31)	1.92 (0.28, 15.14)	2.22 (0.24, 16.65)
1.02 (0.25, 5.41)	0.67 (0.12, 4.29)	1.40 (0.22, 12.60)	0.58 (0.08, 4.47)	**Dorsal plating**	1.21 (0.27, 5.39)	1.26 (0.16, 11.65)
0.84 (0.20, 4.06)	0.57 (0.10, 3.51)	1.17 (0.16, 12.40)	0.52 (0.07, 3.57)	0.82 (0.19, 3.65)	**Volar plating**	1.06 (0.13, 9.89)
0.76 (0.14, 4.60)	0.52 (0.10, 2.85)	1.06 (0.20, 6.83)	0.45 (0.06, 4.22)	0.79 (0.09, 6.24)	0.94 (0.10, 7.56)	**Dorsal and volar plating**

Notes: a: carpal tunnel syndrome; b: pin-track infection; c: complex regional pain syndrome.

**Table 3:** The results of surface under the cumulative ranking curve of seven treatment modalities.

Treatment	Outcomes		
	CTS risks	PTI rates	CRPS risks
Bridging external fixation	37.1%	21.1%	51.2%
Non-bridging external fixation	49.3%	0.4%	72.3%
K-wire fixation	24.1%	39.1%	24.8%
Plaster fixation	51.9%	75.3%	77.3%
Dorsal plating	89.4%	68.2%	47.5%
Volar plating	40.2%	72.6%	43.5%
Dorsal and volar plating	57.9%	73.4%	33.4%

Notes: CTS, carpal tunnel syndrome; PTI, pin-track infection; CRPS: complex regional pain syndrome.
